# Comparative evaluation of ^68^Ga-labelled TATEs: the impact of chelators on imaging

**DOI:** 10.1186/s13550-020-00620-6

**Published:** 2020-04-15

**Authors:** Yuxiao Xia, Chengrun Zeng, Yanhong Zhao, Xinyi Zhang, Zibo Li, Yue Chen

**Affiliations:** 1 Department of Nuclear Medicine, The Affiliated Hospital of Southwest Medical University, No. 25, Taiping St, Luzhou, 646000 Sichuan People’s Republic of China; 2 Nuclear Medicine and Molecular Imaging Key Laboratory of Sichuan Province, No. 25, Taiping St, Luzhou, 646000 Sichuan People’s Republic of China; 3 Biomedical Research Imaging Center, Department of Radiology, and UNC Lineberger Comprehensive Cancer Center, University of North Carolina-Chapel Hill, Chapel Hill, NC 27514 USA

**Keywords:** ^68^Ga-NOTA-TATE, ^68^Ga-DOTA-TATE, Bifunctional chelating agent, Neuroendocrine tumour, AR42J tumour-bearing mouse, Pharmacokinetics, Positron emission tomography, Micro-PET/CT, Healthy human imaging, Maximum standardized uptake values

## Abstract

**Background:**

^68^Ga-labelled peptides targeting somatostatin receptor 2 (SSTR2) have demonstrated encouraging results in managing patients with neuroendocrine tumours (NETs). In addition to metal chelation, bifunctional chelators have also been found to impact imaging outcomes due to their differences in stability, charge, hydrophilicity, etc. In the present work, a comparative pharmacokinetic evaluation and imaging characteristics were performed between ^68^Ga-labelled somatostatin analogues (TATE) using NOTA (1,4,7-triazacyclononane-1,4,7-triacetic acid) and DOTA (1,4,7,10-tetraazacyclododecane-1,4,7,10-tetraacetic acid) as bifunctional chelating agents (BFCAs).

**Results:**

Both ^68^Ga-NOTA-TATE and ^68^Ga-DOTA-TATE were obtained with high radiochemical purity. ^68^Ga-NOTA-TATE demonstrated higher in vitro stability (≥ 99%) than ^68^Ga-DOTA-TATE (≥ 95%) after 3 h of incubation. The water solubilities (partition coefficients, − 1.76 ± 0.06 vs. − 2.72 ± 0.16) and plasma protein binding rates (12.12% vs. 30.6%) were lower for ^68^Ga-NOTA-TATE than for ^68^Ga-DOTA-TATE. Differential pharmacokinetics and comparable tumour affinities (within 1 h) were observed in AR42J tumour-bearing mice. Healthy volunteer imaging studies showed comparable distribution patterns of these two imaging agents. However, the maximum standardized uptake values (SUVmax) of the two tracers varied in each organ. The two PET agents demonstrated almost identical SUVmax values in the kidneys. ^68^Ga-NOTA-TATE did have a lower SUVmax in most other organs compared with ^68^Ga-DOTA-TATE, including the liver (4.2 vs. 10.1), potentially due to the lower protein binding rate.

**Conclusion:**

^68^Ga-NOTA-TATE and ^68^Ga-DOTA-TATE demonstrated comparable tumour uptake in an AR42J mouse model. An initial clinical study revealed that ^68^Ga-NOTA-TATE may have reduced background uptake in the major organs such as the liver. Although the subject numbers were limited, further investigation of ^68^Ga-NOTA-TATE is warranted for detecting SSTR2-positive neuroendocrine tumours.

## Background

Neuroendocrine tumours (NETs) are a group of malignancies that originate from neuroendocrine cells. Most of the primary lesions of NETs are located in the digestive tract and respiratory tract and usually grow slowly but can cause significant morbidity and mortality. In the USA, the incidence of NETs has increased more than six-fold in the past few decades [1], and NETs have a higher prevalence (112,000 cases) than gastric adenocarcinoma or pancreatic malignancies [2]. Although NETs are considered to be a relatively benign disease, these tumours are highly metastatic and often involve multiple metastases throughout the body. It is essential to accurately delineate the extent of the disease for proper management. Because neuroendocrine tumours can express high levels of the somatostatin receptor, radiolabelled peptides targeting this receptor have shown excellent accuracy in detecting SSTR-positive NETs using positron emission tomography/computed tomography (PET/CT) [3]. Indeed, PET/CT scans using ^68^Ga-labelled somatostatin analogues have become the standard diagnostic practice for NETs under the current guidelines [4, 5], which play a vital role in patient management [6, 7]. In particular, ^68^Ga-DOTA-TATE is a PET agent that was approved by the US Food and Drug Administration (FDA) in 2016 for detecting SSTR-positive NETs. Although DOTA (1,4,7,10-tetraazacyclododecane-1,4,7,10-tetraacetic acid) and NOTA (1,4,7-triazacyclononane-1,4,7-triacetic acid) are two widely used bifunctional chelating agents (BFCAs) for ^68^G a[8], NOTA has been reported as a ^68^Ga chelator with higher stability and milder labelling conditions compared with DOTA. An experimental study reported showed that even in the presence of excess other metal ion impurities, NOTA-NCS (p-isothiocyanato benzyl-1,4,7-triazacyclononane-1,4,7-triacetic acid) could be radiolabelled instantly with ^68^Ga at room temperature with > 98% yield [9]. The ^68^Ga-complex of NOTA-NCS still has high in vitro stability, and ^68^Ga-NOTA-NCS could be cleared rapidly through the kidneys with minimum retention in any major organ [9]. The above study revealed that NOTA has superior complexation efficiency and better pharmacokinetic properties over other BFCAs, including DOTA, upon radiolabelling with ^68^Ga [9]. However, when the BFCAs are attached with biomolecules or other carrier moieties, the pharmacokinetic advantages exhibited by ^68^Ga-NOTA derivatives over ^68^Ga-DOTA derivatives may or may not have any significant impact on the pharmacokinetics and target specificity of radiolabelled agents [8]. Herein, we perform a side-by-side comparison of the biological properties, pharmacokinetics and imaging characteristics of ^68^Ga-labelled NOTA-TATE and DOTA-TATE. Our initial studies warrant further investigation of ^68^Ga-NOTA-TATE as a suitable PET agent for NET diagnosis due to the reduced background.

## Methods

### Materials

^68^Ga was obtained by eluting a ^68^Ge/^68^Ga generator (1850 MBq ^68^Ge/^68^Ga generator, ITG, Germany) with 0.05 M HCl. DOTA-TATE was purchased from ABX advanced biochemical compounds GmbH. The bis-t-butyl NOTA ligand was provided by Immunomedics, Inc. Throl resin and the protected amino acids were purchased from CreoSalus Inc. (Louisville, KY). All other chemicals were purchased from Sigma-Aldrich (St. Louis, MO) or Fisher Scientific (Pittsburgh, PA). A liquid chromatography mass spectrometer (Shimadzu, Inc., Japan) was used to analyse NOTA-TATE. The radiochemical purity was documented using HPLC (LabAlliance, SSI, Inc., USA). A gamma counter (SN-695B; Hesuo Rihuan Photoelectric Instrument Co., Shanghai, China) and a calibrator (CRC-15R; Capintec, Inc., Florham Park, NJ, USA) were used to measure the radioactivity of the samples. Human blood serum and human plasma were provided by the in vitro analysis laboratory of the Department of Nuclear Medicine of the Affiliated Hospital of Southwest Medical University. Scintigraphy was performed by PET/CT (Gemini TF/16, Philips, Netherlands) or micro-PET/CT (Siemens, Germany, Inveon MM gantry, serial number 3125). The AR42J cell line was purchased from Nanjing Kehao Biotechnology Co., Ltd. BALB/c nude mice were purchased from Beijing HFK Bioscience Co., Ltd. Kunming (KM) mice were also purchased (Animal Experiment Center [animal licence SCXK 2013-17], Southwest Medical University, Luzhou, Sichuan, China). All studies were approved by the Ethics Committee of Southwest Medical University.

### Preparation of NOTA-TATE complexes

**Fig. 1:**
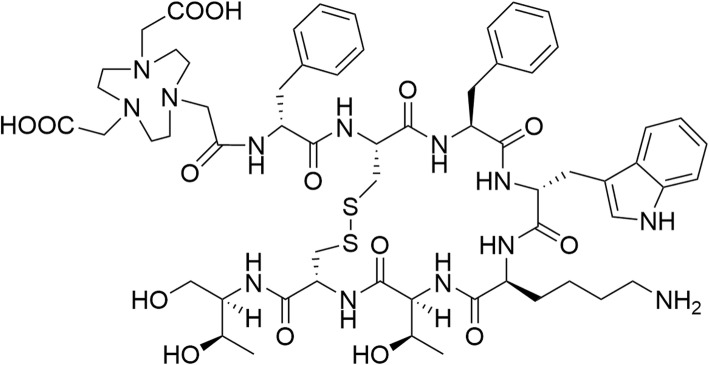
The chemical structure of NOTA-TATE

The octreotide peptide analogue IMP466, NOTA-D-Phe-cyclo[Cys-Phe-D-Trp-Lys-Thr-Cys]-Throl (MH+1305), was synthesized using standard Fmoc-based solid phase peptide synthesis. Throl resin was mixed with 4 equivalents of each Fmoc protected amino acid and 3.9 equivalents of HATU (1-[Bis(dimethylamino)methylene]-1H-1,2,3-triazolo[4,5-b]pyridinium 3-oxid hexafluorophosphate) as the coupling reagent in DMF. After incubation for 4 h, the resin was washed with DMF 3 times and added to a mixture of 96:2:2 DMF/piperidine/DBU (v/v/v) for Fmoc deprotection. After 5 min of incubation, the solvent was removed, and the coupling step was repeated sequentially according to the desired peptide sequence. The peptide was cleaved from the resin with a CH_2_Cl_2_/acetic acid/trifluoroethanol solution (3:1:1 v/v/v) under mild stirring for 2 h. After the peptide was cleaved from the resin, the peptide was cyclized by overnight incubation with DMSO at room temperature. All buffers used for radiolabelling were metal-free. We determined the purity and structural confirmation analysis of precursor NOTA-TATE on a Shimadzu LC-MS, and the results are shown in Supplementary Materials 1. The chemical structure of NOTA-TATE is shown in Fig. 1.

### Preparation of ^68^Ga-labelled NOTA-TATE and DOTA-TATE

In this experiment, NOTA-TATE and DOTA-TATE solutions (20 μg/10 μL), ^68^Ga solution (10–15 mCi/mL) and sodium acetate buffer (pH = 5.5) were prepared. The effects of the corresponding conjugate concentration, ^68^Ga activity, pH, temperature and reaction time on the radiochemical yield and purity of ^68^Ga-labelled NOTA-TATE and DOTA-TATE were investigated by independent reaction conditions. For comparing the distribution and imaging experiments of ^68^Ga-NOTA-TATE and ^68^Ga-DOTA-TATE, both were obtained under the following conditions: The amount of NOTA-TATE or DOTA-TATE was fixed at 20 μg, and the activity of ^68^GaCl_3_ varied (370 MBq-555 MBq in 1 mL of 0.05 M HCl). The pH of respective mixtures was adjusted by adding sodium acetate buffer. The pH of the mixture was adjusted to 3.5–4, and ^68^Ga-NOTA-TATE was obtained by incubating at 90 °C for 10 min. ^68^Ga-DOTA-TATE was obtained by adjusting the pH of the mixture to 4–4.5 and incubating at 90 °C for 10 min. The radiolabelled conjugates were purified using preconditioned C-18 reversed-phase Sep-Pak cartridges.

### Quality control techniques

Characterization of the radiolabelled complexes was carried out using reversed-phase HPLC. Water (A) and acetonitrile (B) each mixed with 0.1% trifluoroacetic acid was used as the mobile phase with gradient elution (0–3 min, 0–10% B; 3–15 min, 10–70% B; 15–20 min, 70–10% B) to separate the free ^68^Ga from the ^68^Ga complexes. The elution was monitored by detecting the radioactivity signal using an NaI (Tl) detector and the UV signal at 254 nm. The flow rate was maintained at 1 mL/min for elution.

### Serum binding and in vitro stability studies

In vitro serum binding studies for both ^68^Ga-labelled NOTA-TATE and DOTA-TATE were carried out by following a generalized procedure summarized below. One millilitre of fresh anticoagulated (heparinized) human plasma was prepared. Freshly labelled compound (3.7 MBq, 0.1 mCi) was added to three centrifuge tubes, each containing 0.1 mL of human plasma. After incubation at 37 °C for 2 h, 1 mL of ice-cold methanol was added to each tube. The mixtures were separated by centrifugation (5 min, 3000 rpm) to collect the supernatant, which was repeated three times. A gamma counter was then used to measure the radioactivity of the supernatant (A) and the precipitate (B) in counts per minute (CPM); the plasma-protein binding rate was calculated as PPB = B/(A + B)100%, and the average of three tubes was recorded.

To evaluate the in vitro stability, the radiolabelled preparation was incubated with human blood serum in a water bath at a constant temperature of 37 °C. Aliquots (100 μL) were taken from the reaction mixture at 15 min, 30 min, 1 h, 2 h, 3 h and 4 h, followed by precipitation of serum proteins using acetonitrile. The solution mixture was centrifuged, and the supernatant was analysed by HPLC using the protocol mentioned above. In addition, we added the labelled formulations to physiological saline and incubated them at 37 °C. All the labelled compounds were tested by HPLC for radiochemical purity at each of the abovementioned time points.

### Determination of the lipid-water partition (log P_o/w_)

The lipid-water partition (logP) of ^68^Ga-labelled complexes was determined in the octanol water system by following the procedure mentioned below. In brief, 490 μL of ultrapure water was added to each of three centrifuge tubes containing 0.5 mL of saturated n-octanol and then combined with 10 μL (1.85 MBq/0.05 mCi) of freshly prepared radiolabelled complexes by ultrasonication (3 min). The centrifuge tube was allowed to stand for approximately 1 min for the liquid to become stratified. The upper and lower liquids were defined as groups A (A1, A2, A3) and B (B1, B2, B3), accounting for the organic and aqueous phases, respectively. Then, 0.1 mL was retrieved from each of the 6 sections. The radioactivity (CPM) of both phases was determined using a gamma counter, and the lipid-water partition coefficient was calculated as follows: logP = log (A-background)/(B-background). The average of three tubes was recorded as the logP.

### Biodistribution and imaging studies in AR42J pancreatic tumour xenografts

Tumour cells were subcutaneously injected into the left forelimb of each nude mouse. Biodistribution and imaging studies were conducted when the tumour volume reached at least 1.0 × 1.0 × 1.0 cm^3^.

The biodistribution of ^68^Ga-NOTA-TATE in AR42J tumour-bearing mice (weight range, 15–20 g) was studied. Each mouse was injected with 0.1 mL of ^68^Ga-NOTA-TATE (0.1 mCi, 8.8 GBq/mg) in the tail vein. At 15 min, 30 min, 1 h and 2 h after injection, groups of four tumour-bearing mice (2 females and 2 males) were sacrificed by cervical dislocation to determine the percent injected dose per gramme (%ID/g) in various samples of mouse tissues (i.e. the blood, liver, spleen, lung, kidney, gallbladder, stomach, intestine, femur, muscle and tumour) with a gamma counter. For comparison, biodistribution studies of ^68^Ga-DOTA-TATE were performed the same way.

Six (3 female and 3 male) mice with AR42J tumour xenografts (15–20 g) were used in the imaging study. All tumour-bearing mice were anaesthetized by intranasal inhalation of isoflurane. Each mouse was immediately injected with 0.1 mL of ^68^Ga-NOTA-TATE or ^68^Ga-DOTA-TATE at a dose of 2.96 MBq (0.08 mCi). Images were acquired and reconstructed using micro-PET/CT at 30 min and 2 h after intravenous injection.

### Acute animal toxicity test of ^68^Ga-NOTA-TATE

Thirty KM mice were randomly divided into a control group, a ^68^Ga- NOTA-TATE low-dose group (100 μCi) and a high-dose group (1 mCi). Each group contained 10 mice, half female and half male. The dose of the appropriate compound was administered through the tail vein at an administration volume of 0.2 mL. The animal blood routine, body weight and animal feed consumption were performed before administration and on days 4, 7, 14, 21 and 28 after administration. At the end of the observation period, the surviving mice were dissected, and histopathological examination was performed on the myocardium, liver, spleen, lung, kidney, stomach, intestine, gonad, bone, muscle and brain of each mouse.

### Volunteer imaging study

Twenty-two healthy volunteers gave informed consent and agreed to participate in the study. Twelve volunteers (6 males and 6 females, age 47.3 ± 12.1 years) underwent ^68^Ga-NOTA-TATE PET/CT scans, and ten volunteers (5 males and 5 females, age 42.9 ± 14.7 years) underwent ^68^Ga-DOTA-TATE PET/CT scans. All volunteers did not have a history of treatment with cold octreotide and did not fast before imaging. Each patient was injected with an average activity of 1.9 MBq/kg. The timing of image acquisition ranged from 30 to 45 min after injection (3 min/bed position). The PET image was attenuated and then reconstructed using the iterative reconstruction algorithm implemented.

## Results

### Radiolabelling and quality control

**Fig. 2:**
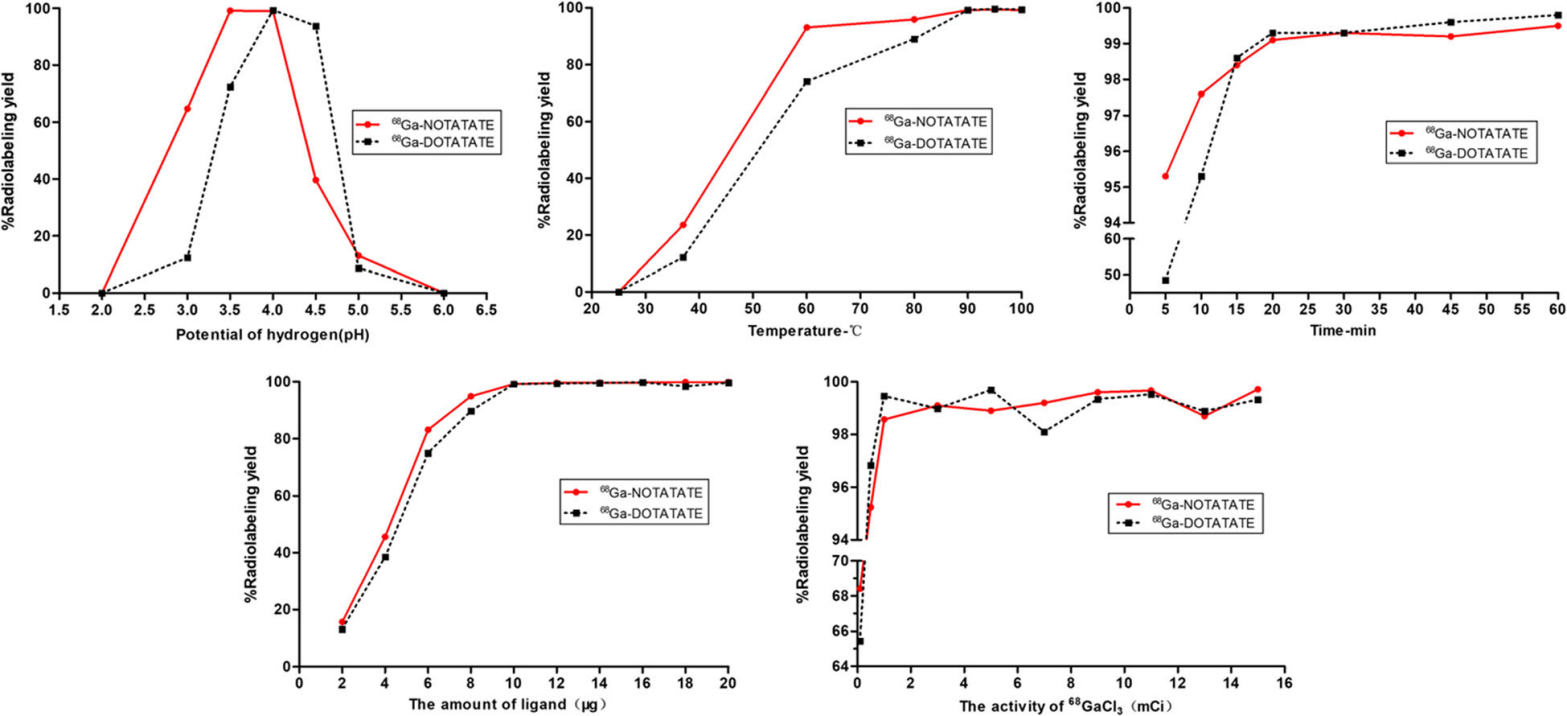
The factor effects on the radiochemical purity of ^68^Ga-NOTA-TATE and ^68^Ga-DOTA-TATE

The effects of pH, temperature, reaction time, activity of ^68^GaCl_3_ and the amount of NOTA-TATE and DOTA-TATE on the labelling rate are shown in Fig. 2. The pH had a great effect on ^68^Ga-labelled NOTA-TATE (optimal pH = 3.5–4) and DOTA-TATE (optimal pH = 4–4.5). Under the optimal pH conditions, ^68^Ga-NOTA-TATE can be prepared at a lower temperature and in a shorter time. The molar activities of ^68^Ga-NOTA-TATE and ^68^Ga-DOTA-TATE obtained by labelling were 8.8 GBq/mg and 7.4 GBq/mg, respectively. HPLC analysis of the two complexes showed ≥ 99.0% radiochemical purity. The retention time of ^68^Ga-NOTA-TATE in the optimized HPLC system was 12.1 ± 0.05 min (*n* = 12), and the retention time of ^68^Ga-DOTA-TATE in the same gradient solvent system was 11.8 ± 0.08 min (*n* = 10) (radiochromatograms can be found in the Supplementary Materials 2).

### Characterization of the ^68^Ga-labelled NOTA-TATE and DOTA-TATE complexes

**Fig. 3:**
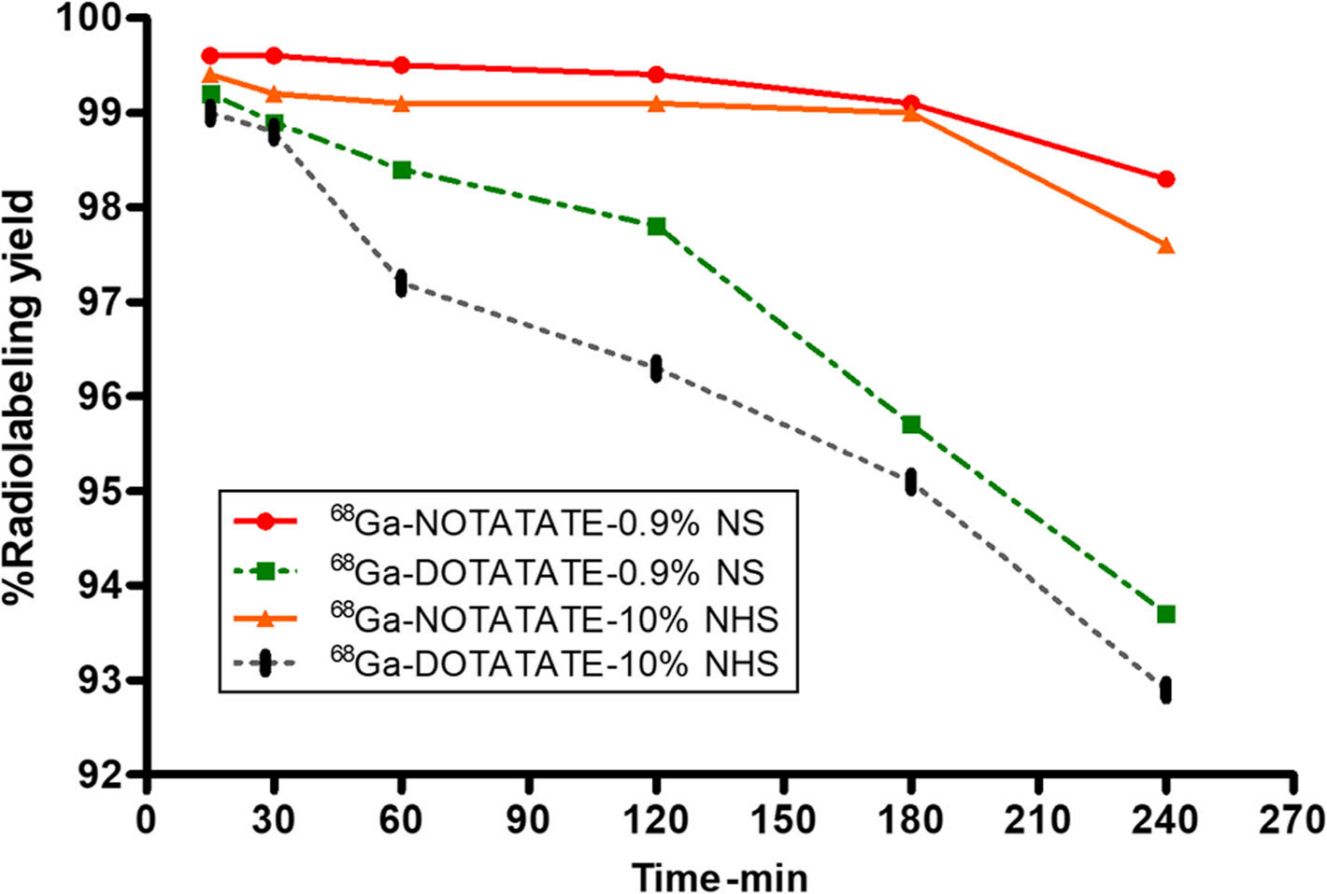
The in vitro stabilities under different conditions of ^68^Ga-NOTA-TATE and ^68^Ga-DOTA-TATE

The log P_o/w_ values for ^68^Ga-NOTA-TATE and ^68^Ga-DOTA-TATE were found to be − 1.76 ± 0.06 and − 2.72 ± 0.16, respectively. These values indicate that both radiolabelled complexes are hydrophilic in nature and that ^68^Ga-DOTA-TATE is more hydrophilic than ^68^Ga-NOTA-TATE. The radiolabelled conjugates revealed serum binding percentages of 12.12% and 30.6%, respectively, indicating that ^68^Ga-DOTA-TATE has a stronger affinity for serum proteins than ^68^Ga-NOTA-TATE. We also attempted to determine the in vitro stability of the two radiolabelled complexes (Fig. 3) and found that the radiolabelled conjugates maintained high radiochemical purity in physiological saline or human serum (> 99% for ^68^Ga-NOTA-TATE and > 95% for ^68^Ga-DOTA-TATE after 3 h of incubation).

### Imaging and biodistribution studies in AR42J pancreatic tumour xenografts

The biodistribution of ^68^Ga-NOTA-TATE and ^68^Ga-DOTA-TATE in nude mice bearing AR42J tumours is shown in Table 1. The radioactivity of ^68^Ga-NOTA-TATE in the gallbladder was significantly higher than that of ^68^Ga-DOTA-TATE, suggesting that ^68^Ga-NOTA-TATE can be excreted through the biliary system in mice. The radioactivity of ^68^Ga-NOTA-TATE in the spleen was significantly lower than that of ^68^Ga-DOTA-TATE. In animal experiments, the liver showed no significant difference in radioactivity uptake. Both complexes had high kidney uptake and low uptake in the bones and muscles. In the lungs, stomach and intestines, the activity of ^68^Ga-NOTA-TATE was slightly lower than that of ^68^Ga-DOTA-TATE. The radioactivity in the blood cleared quickly with time. After 1 h of intravenous injection, the blood activity of ^68^Ga-NOTA-TATE and ^68^Ga-DOTA-TATE was only 0.58 ± 0.24% ID/g and 0.35 ± 0.20% ID/g, respectively. The tumour tissue had prominent uptake of both octreotide complexes. After intravenous injection of ^68^Ga-NOTA-TATE and ^68^Ga-DOTA-TATE, there was no significant difference in the radioactivity counts of the two compounds in tumour tissues: 15 min (3.24 ± 1.03% ID/g vs. 3.13 ± 1.75% ID/g), 30 min (3.97 ± 2.24% ID/g vs. 3.62 ± 1.94% ID/g) and 1 h (3.16 ± 1.92% ID/g vs. 3.14 ± 2.07% ID/g), respectively. After 2 h, the radioactivity count of ^68^Ga-NOTA-TATE (1.33 ± 0.96% ID/g) in tumour tissue was lower than that of ^68^Ga-DOTA-TATE (2.3 ± 1.34% ID/g).

**Table 1:** Biodistribution of ^68^Ga-NOTA-TATE and ^68^Ga-DOTA-TATE in percentage of injected dose per gramme of organ at 15 min, 0.5 h, 1 h and 2 h postinjection in mice bearing AR42J tumours

%ID/g of organ or tissue								
Organ/tissue	^68^Ga-DOTA-TATE				^68^Ga-NOTA-TATE			
	15 min	0.5 h	1 h	2 h	15 min	0.5 h	1 h	2 h
Blood	3.03 ± 1.96	1.32 ± 0.39	0.35 ± 0.20	0.05 ± 0.02	3.25 ± 1.06	1.01 ± 0.42	0.58 ± 0.24	0.07 ± 0.06
Liver	1.27 ± 0.77	0.61 ± 0.35	0.34 ± 0.25	0.08 ± 0.07	1.19 ± 0.54	0.43 ± 0.23	0.07 ± 0.04	0.04 ± 0.02
Spleen	3.84 ± 4.03	1.48 ± 0.84	1.92 ± 1.75	0.21 ± 0.19	0.19 ± 0.08	0.13 ± 0.11	0.12 ± 0.06	0.03 ± 0.01
Lungs	5.10 ± 5.22	2.86 ± 1.47	4.62 ± 3.42	0.98 ± 0.22	3.64 ± 1.56	0.21 ± 0.17	0.64 ± 0.41	0.48 ± 0.21
Kidney	10.35 ± 8.89	8.12 ± 2.71	2.82 ± 2.54	1.49 ± 0.57	17.1 ± 6.39	4.23 ± 2.73	3.79 ± 2.16	1.69 ± 1.02
Gallbladder	1.47 ± 0.62	0.79 ± 0.48	0.52 ± 0.31	0.11 ± 0.06	3.62 ± 1.94	6.21 ± 2.69	1.83 ± 0.84	1.61 ± 0.67
Stomach	4.24 ± 2.66	0.98 ± 0.49	4.26 ± 2.58	0.52 ± 0.39	0.19 ± 0.13	3.55 ± 1.83	1.61 ± 1.03	1.11 ± 0.21
Intestines	1.56 ± 1.47	1.58 ± 1.77	0.91 ± 0.52	0.23 ± 0.15	0.07 ± 0.04	0.03 ± 0.02	0.21 ± 0.09	0.13 ± 0.07
Skeleton	1.94 ± 0.81	1.10 ± 0.63	0.80 ± 0.73	0.17 ± 0.06	2.14 ± 1.41	1.46 ± 0.37	0.85 ± 0.38	0.13 ± 0.07
Muscle	1.18 ± 1.26	0.60 ± 0.45	0.07 ± 0.02	0.04 ± 0.01	0.26 ± 0.03	0.47 ± 0.26	0.08 ± 0.06	0.03 ± 0.01
Tumour	3.13 ± 1.75	3.62 ± 1.94	3.14 ± 2.07	2.3 ± 1.34	3.24 ± 1.03	3.97 ± 2.24	3.16 ± 1.92	1.33 ± 0.96
Tumour/kidney	0.30	0.45	1.11	1.54	0.18	0.94	0.81	0.79
T/NT	2.65	6.03	44.86	57.50	12.46	8.45	39.5	44.3

*T/NT* tumour/muscle

**Fig. 4:**
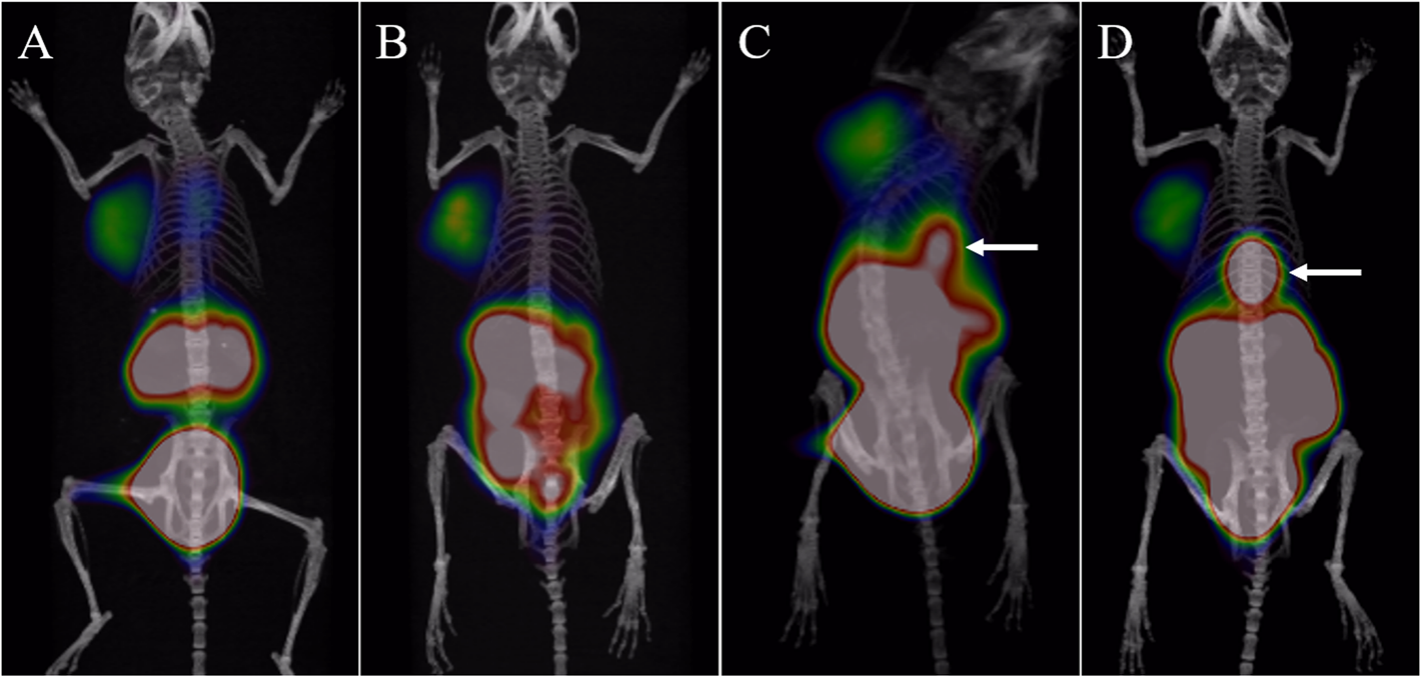
^68^Ga-DOTA-TATE 3D micro-PET/CT imaging at 30 min (**a**, SUVmax = 2.87) and 120 min (**b**, SUVmax = 1.83) in nude mice bearing AR42J tumours. ^68^Ga-NOTA-TATE 3D micro-PET/CT imaging at 30 min (**c**, SUVmax = 3.02) and 120 min (**d**, SUVmax = 1.64) in nude mice bearing AR42J tumours. Compared with ^68^Ga-DOTA-TATE, ^68^Ga-NOTA-TATE imaging showed intense radioactivity in the gallbladder (arrow)

Micro-PET/CT imaging of the AR42J tumour-bearing mice is shown in Fig. 4. The tumours are all clearly visible. ^68^Ga-NOTA-TATE imaging showed that the gallbladder had intense radioactivity, while ^68^Ga-DOTA-TATE imaging showed minimal gallbladder activity.

### Acute animal toxicity test of ^68^Ga-NOTA-TATE

After ^68^Ga-NOTA-TATE was injected into the tail vein of mice, the body weights of the animals in each administration group were not significantly different from that of the control group (*P* > 0.05), and the trend of feed consumption was comparable. The results of histopathological examination showed that the agent had no toxic pathological changes associated with the test substance. The WBC, PLT and RBC counts were all within the normal range.

### Volunteer imaging study

**Fig. 5:**
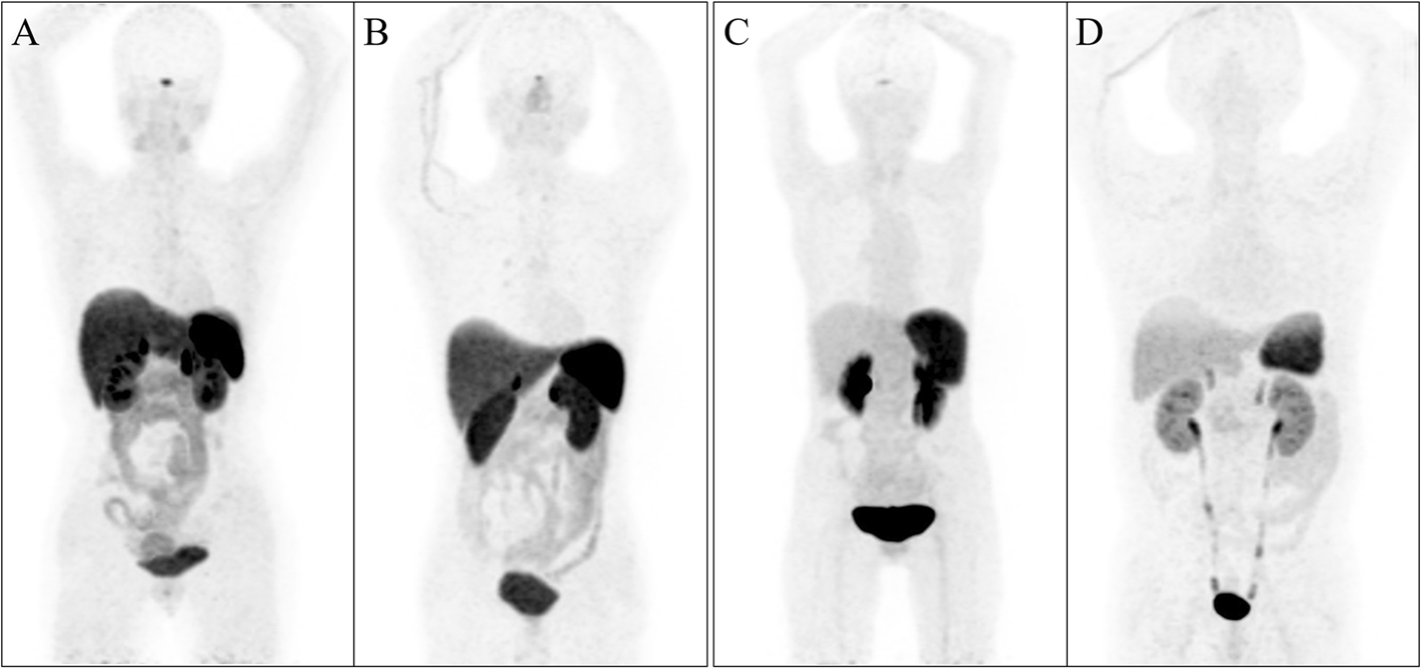
Representative maximum-intensity PET/CT projection images of ^68^Ga-DOTA-TATE (**a**, **b**) and ^68^Ga-NOTA-TATE (**c**, **d**) in healthy volunteers 35–45 min after injection. **a** Female, 46 years old, 51 kg. Maximum intensity projection image after intravenous injection of ^68^Ga-DOTA-TATE (2.52 mCi) (pituitary, SUVmax = 7.74; spleen, SUVmax = 25.5; liver, SUVmax = 9.4; kidney, SUVmax = 13.9). **b** Male, 37 years old, 58 kg. Maximum intensity projection image after intravenous injection of ^68^Ga-DOTA-TATE (2.80 mCi) (pituitary, SUVmax = 4.2; spleen, SUVmax = 16.3; liver, SUVmax = 7.8; kidney, SUVmax = 10.5). **c** Female, 35 years old, 33 kg. Maximum intensity projection image after intravenous injection of ^68^Ga-NOTA-TATE (1.86 mCi) (pituitary, SUVmax = 1.9; spleen, SUVmax = 6.8; liver, SUVmax = 1.4; kidney, SUVmax = 12.5). **d** Male, 57 years old, 83 kg. Maximum intensity projection image after intravenous injection of ^68^Ga-NOTA-TATE (3.83 mCi) (pituitary, SUVmax = 0.7; spleen, SUVmax = 3.9; liver, SUVmax = 1.3; kidney, SUVmax = 6.2). Compared with ^68^Ga-DOTA-TATE, ^68^Ga-NOTA-TATE imaging provides a potential significant advantage in detecting lesions in the liver region due to the distinctively lower liver background

Representative maximum-intensity projection images of the two radioactive compounds are shown in Fig. 5. The range and average values of the maximum standardized uptake values (SUVmax) of ^68^Ga-NOTA-TATE (*n* = 12) and ^68^Ga-DOTA-TATE (*n* = 10) in the major organs of healthy volunteers are shown in Table 2. Moving from the brain to the thigh root, the following observations can be made regarding the distribution of the radiopharmaceuticals. Organs with high expression levels of somatostatin receptor 2 (SSTR2), such as the pituitary gland, submandibular salivary gland, thyroid, adrenal gland, liver and spleen, exhibited high ^68^Ga-DOTA-TATE activity in vivo. In the head and neck region, we observed that ^68^Ga-DOTA-TATE was not taken up in the brain. It should be noted that ^68^Ga-DOTA-TATE can be taken up by the pituitary gland (SUVmax = 7.9). Generally, ^68^Ga-DOTA-TATE can be diffusely taken up by the thyroid gland over a wide range (SUVmax = 1.5–5.9). In the chest region, we found that the level of ^68^Ga-DOTA-TATE uptake in lung tissue was low (SUVmax = 1.3), suggesting that the expression of SSTR2 in lung tissue was low. In the breast, ^68^Ga-DOTA-TATE mainly showed diffuse low uptake in glandular tissues (SUVmax = 0.4–0.9). In the abdominal region, we observed strong uptake of ^68^Ga-DOTA-TATE in the spleen (SUVmax = 33.5), which is related to the high expression of SSTR2 in splenic tissue. Diffuse high uptake of ^68^Ga-DOTA-TATE (SUVmax = 7.8–12.0) was observed in the liver. No radioactivity was seen in the gallbladder, indicating that ^68^Ga-DOTA-TATE is not excreted through the biliary system. ^68^Ga-DOTA-TATE was taken up in the pancreatic tissue at a moderate level, with a large range (SUVmax = 3.1–8.3), and the distribution was not uniform. ^68^Ga-DOTA-TATE was taken up at a moderate to high level in the gastrointestinal tract, and ^68^Ga-DOTA-TATE was relatively higher taken up by the stomach wall (SUVmax = 8.3). The adrenal glands could significantly take up ^68^Ga-DOTA-TATE (SUVmax = 17.7). ^68^Ga-DOTA-TATE is a hydrophilic compound that is mainly excreted by the urinary system. The distal renal tubules and collecting ducts of normal kidneys highly express somatostatin receptors, so both kidneys showed high radioactive activity (SUVmax = 17.8). Lymph nodes can take up ^68^Ga-DOTA-TATE (SUVmax = 0.5–2.0). The physiological distribution of ^68^Ga-NOTA-TATE in healthy volunteers is similar to that of ^68^Ga-DOTA-TATE. However, ^68^Ga-NOTA-TATE and ^68^Ga-DOTA-TATE have significant differences in the degree of uptake in some tissues and organs. ^68^Ga-NOTA-TATE demonstrated a lower average SUVmax in the pituitary gland (1.5 vs. 4.9), salivary glands (parotid gland (0.9 vs. 2.7), submandibular (1.2 vs. 3.4) and palatine tonsil (1.3 vs. 2.6)), thyroid (1.7 vs. 3.8), liver (4.2 vs. 10.1), spleen (14.6 vs. 26.9), pancreas (3.7 vs. 6.2), stomach (2.4 vs. 6.1), small bowel (1.8 vs. 3.4), large bowel (1.5 vs. 2.2) and adrenal glands (10.7 vs. 15.2). The average value of SUVmax in the blood pool was slightly higher for ^68^Ga-NOTA-TATE (1.4 vs. 0.9). The SUVmax of the kidneys (14.1 vs. 14.5) and other organs was comparable with that of ^68^Ga-DOTA-TATE from PET/CT imaging.

**Table 2:** Maximum standardized uptake values (SUVmax) of ^68^Ga-NOTA-TATE and ^68^Ga-DOTA-TATEPET/CT imaging in various organs of healthy volunteers

Organ	^68^Ga-NOTA-TATE (*n* = 12)		^68^Ga-DOTA-TATE (*n* = 10)	
	Average	Range	Average	Range
Pituitary gland	1.5	0.7~2.3	4.9	2.4~7.9
Parotid gland	0.9	0.7~1.3	2.7	1.9~5.4
Submandibular	1.2	0.7~1.4	3.4	1.7~5.7
Palatine tonsil	1.3	0.6~1.8	2.6	1.2~4.1
Thyroid	1.7	1.2~2.5	3.8	1.5~5.9
Lymph nodes	1.2	0.8~1.4	1.1	0.5~2.0
Breast	0.7	0.5~1.1	0.6	0.4~0.9
Thymus	1.1	0.6~1.4	1.2	0.7~1.5
Lungs	0.6	0.3~0.9	0.8	0.5~1.3
Blood pool	1.4	0.9~1.6	0.9	0.6~1.4
Bone marrow	0.9	0.7~1.1	0.8	0.5~1.3
Liver	4.2	1.3~6.3	10.1	7.8~12.0
Spleen	14.6	10.7~17.1	26.9	20.1~33.5
Pancreas	3.7	2.0~5.5	6.2	3.9~8.1
Stomach	2.4	1.6~4.9	6.1	3.1~8.3
Small bowel	1.8	1.4~2.5	3.4	2.2~4.9
Large bowel	1.5	1.0~2.4	2.2	1.6~3.7
Adrenals	10.7	5.7~15.6	15.2	10.8~17.7
Kidneys	14.1	6.2~20.3	14.5	11.2~17.8

## Discussion

With high affinity towards somatostatin receptor subtype 2 (SST2), ^68^Ga-DOTA-TATE plays an important role in NET staging, which often overexpresses SST2 [10]. Compared with DOTA, several studies have reported NOTA as an excellent bifunctional chelating agent (BFCA) for developing ^68^Ga-based PET agents [11, 12]. The cavity size of DOTA is larger than that of NOTA, and the DOTA ^68^Ga complex adopts a pseudo-octahedral geometry with a log stability constant value of 21.33, as reported in the literature [9]. In contrast, NOTA forms a highly stable octahedral complex with ^68^Ga, having a log stability constant value of 30.98 [13]. NOTA can form a ^68^Ga complex not only with high thermodynamic stability but also with adequate kinetic inertness, especially in the pH range of 2–8 and in the presence of cations such as Ca^2+^, Mg^2+^ and Zn^2+^ found in blood serum [9]. DOTA is in common clinical use for neuroendocrine tumour imaging, and the NOTA equivalent has been investigated previously. However, it is unknown whether ^68^Ga-NOTA conjugated somatostatin analogues will exhibit pharmacokinetic advantages over ^68^Ga-DOTA derivatives, and no direct comparison study of this rigour with clinical outputs has been reported. Therefore, in this report, we investigated the effect of BFCAs on the pharmacokinetics, tumour specificity and distribution of ^68^Ga-labelled somatostatin analogues in human subjects.

Compared to ^68^Ga-DOTA-TATE, ^68^Ga-NOTA-TATE can be prepared (pH = 3.5) at a lower temperature (80 °C) and with a shorter incubation time (5 min). Both radiolabelled products have high radiochemical purity and good in vitro stability. The molar activity of ^68^Ga-NOTA-TATE (8.8 GBq/mg) is higher than that of ^68^Ga-DOTA-TATE (7.4 GBq/mg), which is one of the main advantages of NOTA tracers. It may also be a potential source of the differences observed in the PK values of the two tracers. NODAGA (1,4,7-triazacyclononane,1-glutaric acid-4,7-acetic acid) is also a good dual-functional chelating agent for ^68^Ga. ^68^Ga-OPS202 (an SSTR antagonist), also called ^68^Ga-NODAGA-JR11 (JR11 = Cpa-c(_D_Cys-Aph(Hor)-_D_Aph(Cbm)-Lys-Thr-Cys)-_D_Tyr-NH2), is a radiolabelled antagonist that may recognize more SST receptor binding sites [14, 15] and has the potential to improve SST receptor PET/CT imaging. The half inhibitory concentration (IC_50_) of ^68^Ga-OPS202 (antagonist) on SSTR2 is 1.2 ± 0.2, and the half inhibitory concentration (IC_50_) of ^68^Ga-DOTA-TATE (agonist) on SSTR2 is 0.2 ± 0.04, indicating that both have a high affinity for SSTR2 [16]. The reason this study used NOTA instead of NODAGA was due to its widespread commercial availability and use in many previous studies. Both OPS202 and DOTA-TATE have the same number of carboxylate groups, hence having the same overall charge, which is particularly relevant in terms of the effects of logP on the PK. There are four carboxylate groups in DOTA with one used in conjugation compared with three carboxylate groups in NOTA with one used in conjugation. The overall charge of the molecule changes due to the variation in the number of carboxylate groups. This may be the reason for the large difference in the logP values between ^68^Ga-NOTA-TATE and ^68^Ga-DOTA-TATE, which may have a significant impact on the overall properties of each tracer. The ^68^Ga-NOTA complex is neutral, while the ^68^Ga-DOTA complex is negatively charged, which may facilitate faster clearance of the radiotracer through the urinary pathway from the physiological system. This may be the reason why ^68^Ga-labelled NOTA-TATE is less hydrophilic than ^68^Ga-DOTA-TATE and the clearance rate in the blood of tumour-bearing nude mice is also slightly lower. However, studies have shown that ^68^Ga-p-NH_2_-benzyl-NOTA has faster blood clearance and better pharmacokinetic properties than ^68^Ga-p-NH_2_-benzyl-DOTA [9]. It can therefore be inferred that once the BFCAs are conjugated to other molecules, the pharmacokinetic behaviour may be governed by properties of the whole molecule instead of by the properties of the radiolabelled BFCAs. The present study shows that ^68^Ga-labelled TATE prepared using either of these two BFCAs have comparable tumour affinities in nude mice bearing AR42J tumours. In micro-PET/CT imaging studies, the tumour could be clearly visualized, which correlates well with previous reports. Although the retention time of ^68^Ga-NOTA-TATE in tumour tissues seems to be slightly shorter than that of ^68^Ga-DOTA-TATE, the tumour uptake of the two is similar within 1 h, and the tumour can still be clearly visualized after 2 h. The animal experiments indicated that both DOTA and NOTA can be used as suitable BFCAs for preparing ^68^Ga-labelled TATE for PET imaging of neuroendocrine tumour lesions. The acute animal toxicity test of ^68^Ga-NOTA-TATE confirmed that it had no apparent toxicity.

All healthy volunteers showed no apparent side effects after the administration of ^68^Ga-NOTA-TATE. To our knowledge, the distribution pattern of ^68^Ga-DOTA-TATE and the SUVmax in various organs has been documented [17]. In our study, the distribution pattern of ^68^Ga-NOTA-TATE was reported as the range of normal SUVmax in various organs, which was then compared with the SUVmax of ^68^Ga-DOTA-TATE accordingly. No apparent ^68^Ga-NOTA-TATE uptake was noted in the brain, which is similar to that of ^68^Ga-DOTA-TATE. However, in vitro studies found that somatostatin receptor 2 is expressed in the cerebral cortex, limbic system and basal ganglia [17], suggesting that the two radioactive tracers are hydrophilic and not able to cross the blood-brain barrier. In the head and neck region, some glands, such as the pituitary gland, salivary gland and thyroid gland, are not significantly impacted by ^68^Ga-NOTA-TATE, with a relatively low SUVmax. The study found that the expression of somatostatin receptor 2 (SSTR-2) in normal thyroid tissue and thyroid tumour tissue is different [17]. Therefore, the focal uptake of ^68^Ga-NOTA-TATE in the thyroid may have potential significance for the diagnosis of thyroid tumours. There was low ^68^Ga-NOTA-TATE uptake in the lungs, which may be because lung tissue mainly expresses somatostatin receptor 4 (SSTR4). In the blood pool, the SUVmax of ^68^Ga-NOTA-TATE is slightly higher than that of ^68^Ga-DOTA-TATE, which may be related to slower plasma clearance. In the breast, the observed uptake of ^68^Ga-NOTA-TATE was mostly in the glandular tissue and was diffuse and of low grade. In the abdomen, organs with a baseline expression of SSTR2 (such as the spleen, adrenals and kidneys) showed high ^68^Ga-NOTA-TATE uptake in vivo. The uneven spleen imaging pattern may be related to the anatomical distribution of the red and white pulp in the spleen parenchyma [18]. Therefore, the focal spleen uptake of ^68^Ga-NOTA-TATE cannot be misdiagnosed as a tumour. Hepatocytes and hepatic stellate cells are negative for all five somatostatin receptor subtypes [19]. The uptake of the two radioactive tracers by the liver may be because the liver is a metabolic site for peptides. In animal experiments, ^68^Ga-NOTA-TATE had intense radioactivity in the gallbladder of mice, but this was not seen in normal volunteer imaging. It is speculated that this may be due to the difference in the method of excretion of ^68^Ga-NOTA derivatives by mice and humans. The islets can be found in clusters in a pancreatic region since the SST2-positive subtype is located in the islets. Therefore, uneven accumulation of two tracers in the pancreatic region is a normal variant. The SUVmax of ^68^Ga-NOTA-TATE is lower in most other organs, especially in the liver, which is not reflected in the distribution experiments in animals. It is speculated that the reason for the lower SUVmax of ^68^Ga-NOTA-TATE in certain organs may be due to the lower protein binding rate or increased stability. This reduced background may increase the sensitivity towards primary endocrine tumours (such as gastrointestinal neuroendocrine tumours) and metastases (such as liver metastases). Both tracers are hydrophilic and are excreted through the urinary system. Moreover, the distal tubules and collecting ducts of normal kidneys highly express somatostatin receptors, so the bilateral kidneys show high radioactivity. We would like to point out that our study only involved normal volunteers. The observed low background of ^68^Ga-NOTA-TATE in normal organs warrants future evaluation of this agent in NET patients to detect SSTR2-positive lesions.

## Conclusion

In this study, NOTA-TATE was synthesized and radiolabelled with ^68^Ga. Under milder conditions, NOTA-TATE allows for rapid quantitative radiolabelling with higher radiochemical purity and serum stability compared with DOTA-TATE. Biodistribution in the AR42J tumour-bearing nude mice and micro-PET/CT imaging showed that ^68^Ga-NOTA-TATE and ^68^Ga-DOTA-TATE have comparable tumour uptake within 1 h after injection. Healthy volunteer imaging studies showed similar distribution patterns, but the SUVmax of the two tracers varied in each organ. Except for the kidneys, the SUVmax of ^68^Ga-NOTA-TATE was lower than that of ^68^Ga-DOTA-TATE in most organs. This suggests a potential advantage of ^68^Ga-NOTA-TATE in the detection of primary and metastatic foci of neuroendocrine tumours due to the distinctively lower background. This study demonstrates that NOTA can be used in the preparation of ^68^Ga-labelled TATE and has potential for the diagnostic evaluation of neuroendocrine tumour lesions.

## Supplementary information

**Additional file 1: Figure S1.** LC-MS spectra for precursor NOTA-TATE.

**Additional file 2.** Representative HPLC profiles of ^68^Ga-DOTA-TATE (A) and ^68^Ga-NOTA-TATE (B) with retention times of 11.8±0.08 min and 12.1±0.05 min, respectively.

## Data Availability

The datasets used and/or analysed during the current study are available from the corresponding author on reasonable request.
