# Supplementary material for: Comparative evaluation of 68Ga-labelled TATEs: the impact of chelators on imaging
Source: EJNMMI Res. 2020 Apr 15;10:36. doi: 10.1186/s13550-020-00620-6 (PMC7158967; doi:10.1186/s13550-020-00620-6)
Supplement: Supplementary file 1 — Additional file 1: Figure S1. LC-MS spectra for precursor NOTA-TATE. [file 13550_2020_620_MOESM1_ESM.doc]

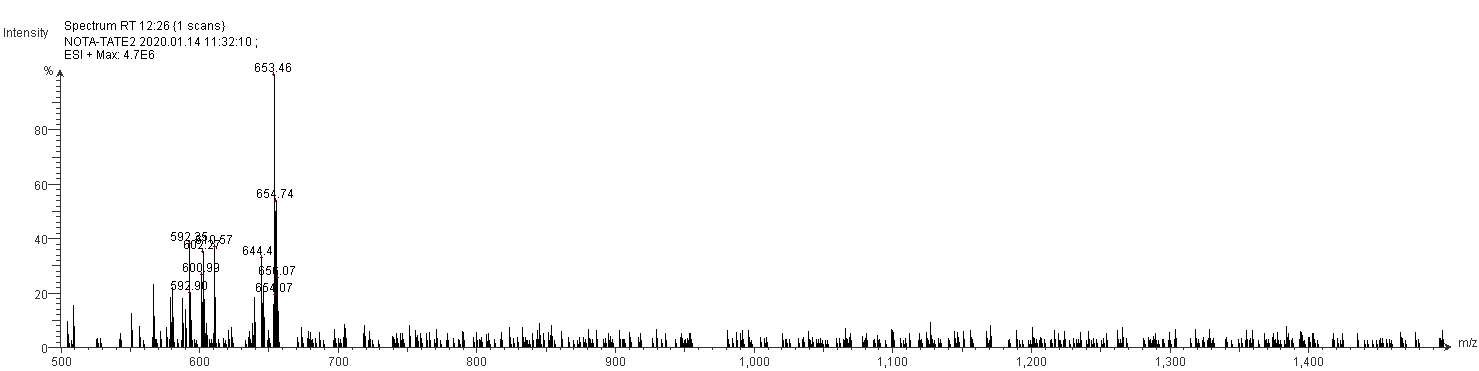


Fig 1. LC-MS spectra for precursor NOTA-TATE.

HPLC conditions: Column: Phenomenex Kinetex 5 μm C18 100A, LC Column 250 × 4.6 mm; Solvent A: 0.1% TFA in water; Solvent B: 0.1% TFA in acetonitrile; Gradient profile: 0 to 2 min, isocratic 5% solvent B; 2 to 22 min, 5-95% solvent B; 22 to 35 min, isocratic 95% solvent B; Flow rate: 1 mL/min; column temperature: 19 to 21°C; UV detector: 212 nm.

We ran the purity and structure confirmation analysis of precursor NOTA-TATE on a Shimadzu LC-MS, and results were as shown in Fig. 1. A single peak was detected at approximately 12 min with a UV detector at 212 nm. The mass spectrometry analysis of this peak showed that the mass-to-charge (m/z) ratio of the compound matched well with the desired product NOTA-TATE (M+2). MS-ESI: [M+2H+] C_61_H_87_N_13_O_15_S_2_ calculated 653.27, found 653.46.
