# Supplementary material for: Comparative evaluation of 68Ga-labelled TATEs: the impact of chelators on imaging
Source: EJNMMI Res. 2020 Apr 15;10:36. doi: 10.1186/s13550-020-00620-6 (PMC7158967; doi:10.1186/s13550-020-00620-6)
Supplement: Supplementary file 2 — Additional file 2. Representative HPLC profiles of 68Ga-DOTA-TATE (A) and 68Ga-NOTA-TATE (B) with retention times of 11.8±0.08 min and 12.1±0.05 min, respectively. [file 13550_2020_620_MOESM2_ESM.docx]

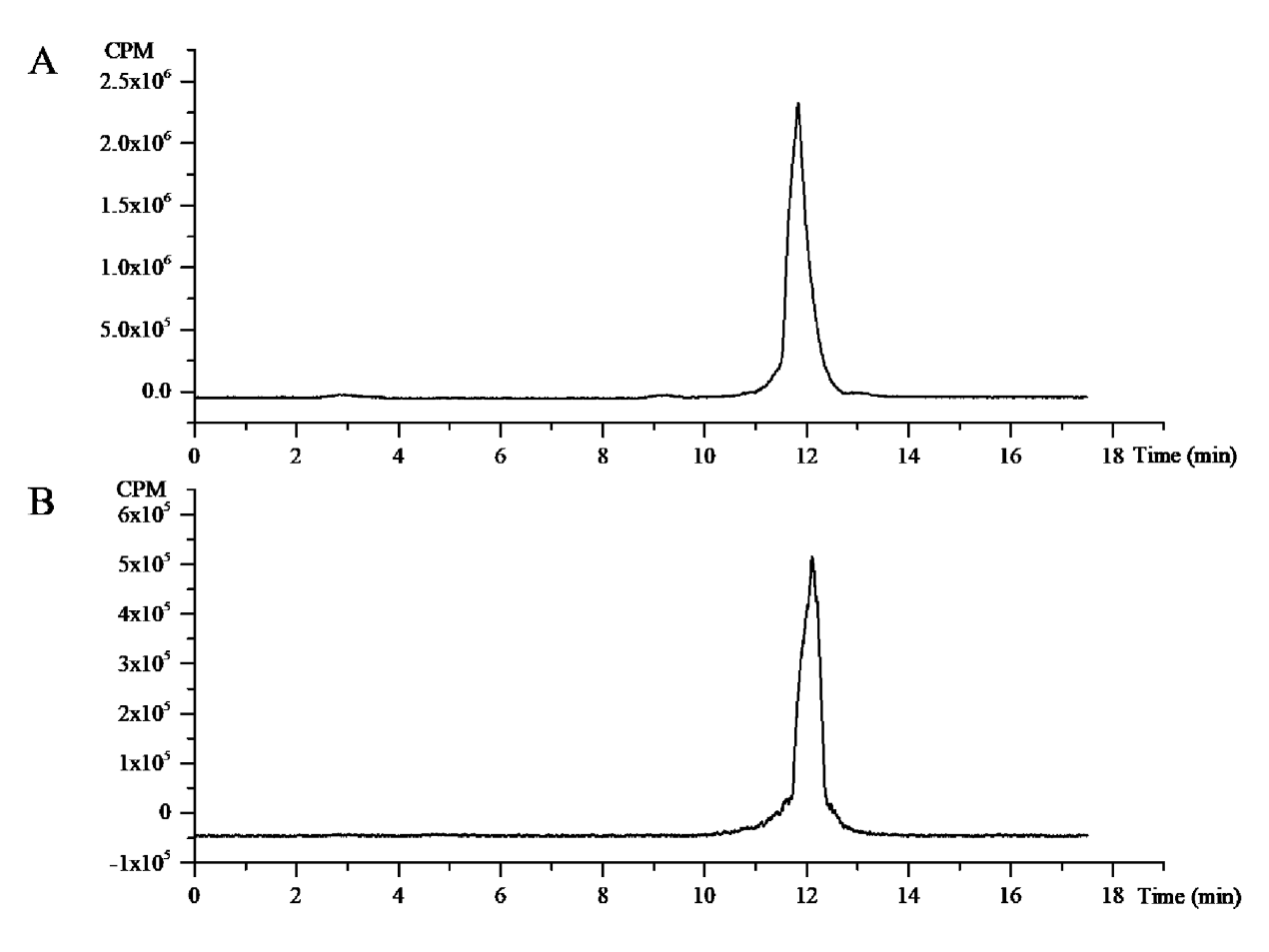
Representative HPLC profiles of ^68^Ga-DOTA-TATE (A) and ^68^Ga-NOTA-TATE (B) with retention times of 11.8±0.08 min and 12.1±0.05 min, respectively.
